# IFITM Proteins Inhibit Entry Driven by the MERS-Coronavirus Spike Protein: Evidence for Cholesterol-Independent Mechanisms

**DOI:** 10.3390/v6093683

**Published:** 2014-09-26

**Authors:** Florian Wrensch, Michael Winkler, Stefan Pöhlmann

**Affiliations:** Infection Biology Unit, German Primate Center, 37077 Göttingen, Germany; E-Mails: fwrensch@dpz.eu (F.W.); mwinkler@dpz.eu (M.W.)

**Keywords:** IFITM, coronavirus, MERS, SARS

## Abstract

The interferon-inducible transmembrane (IFITM) proteins 1, 2 and 3 inhibit the host cell entry of several enveloped viruses, potentially by promoting the accumulation of cholesterol in endosomal compartments. IFITM3 is essential for control of influenza virus infection in mice and humans. In contrast, the role of IFITM proteins in coronavirus infection is less well defined. Employing a retroviral vector system for analysis of coronavirus entry, we investigated the susceptibility of human-adapted and emerging coronaviruses to inhibition by IFITM proteins. We found that entry of the recently emerged Middle East respiratory syndrome coronavirus (MERS-CoV) is sensitive to inhibition by IFITM proteins. In 293T cells, IFITM-mediated inhibition of cellular entry of the emerging MERS- and SARS-CoV was less efficient than blockade of entry of the globally circulating human coronaviruses 229E and NL63. Similar differences were not observed in A549 cells, suggesting that cellular context and/or IFITM expression levels can impact inhibition efficiency. The differential IFITM-sensitivity of coronaviruses observed in 293T cells afforded the opportunity to investigate whether efficiency of entry inhibition by IFITMs and endosomal cholesterol accumulation correlate. No such correlation was observed. Furthermore, entry mediated by the influenza virus hemagglutinin was robustly inhibited by IFITM3 but was insensitive to accumulation of endosomal cholesterol, indicating that modulation of cholesterol synthesis/transport did not account for the antiviral activity of IFITM3. Collectively, these results show that the emerging MERS-CoV is a target of the antiviral activity of IFITM proteins and demonstrate that mechanisms other than accumulation of endosomal cholesterol can contribute to viral entry inhibition by IFITMs.

## 1. Introduction

Coronaviruses are enveloped, positive-sense RNA viruses, which infect birds and mammals [1]. The human coronaviruses (hCoV) 229E, OC43, NL63, and HKU1 are adapted to spread in the human population and circulate globally [1,2]. These viruses cause the common cold, although immunocompromised patients and the elderly occasionally develop more severe disease [3,4,5,6,7,8]. In contrast, the emerging coronaviruses severe acute respiratory syndrome (SARS) coronavirus (SARS-CoV) and Middle East respiratory syndrome (MERS) coronavirus (MERS-CoV) were relatively recently transmitted from bats via intermediate hosts to humans, and can induce severe disease in afflicted patients [9,10,11]. Thus, the SARS outbreak in 2002/2003 was associated with 8096 infections and 774 deaths (case-fatality rate of 9.6%) [12], and the ongoing MERS epidemic, thus far, entails 837 laboratory confirmed infections of which 291 were fatal (as of 23 July 2014), resulting in a case-fatality rate of 35% [13].

The interferon (IFN) response is an integral component of innate immunity against viral infections and the differential pathogenicity of human coronaviruses and emerging coronaviruses might, at least in part, stem from differential susceptibility to inhibition by IFN-induced antiviral effector molecules. The interferon-induced transmembrane proteins (IFITM) 1 to 3 inhibit infection of several enveloped viruses [14,15,16], including hCoV-229E [17] and SARS-CoV [18]. Inhibition usually occurs at the stage of viral entry [14,19], specifically during fusion of the viral membrane with an endosomal membrane [20,21], and might be due to an IFITM-induced accumulation of cholesterol in late endosomes [22]. Expression of IFITM3 is essential for efficient control of influenza A virus (FLUAV) [23,24] and respiratory syncytial virus (RSV) [25] infection in mice and polymorphisms in the IFITM3 locus were found to be associated with the severity of influenza in humans [25]. Although the latter results are controversial [26], these observations indicate that IFITM3 can play an important role in host control of respiratory viruses.

Here, we used vector systems to analyze whether human coronaviruses and emerging coronaviruses are differentially inhibited by IFITM proteins and cationic amphiphiles, which induce accumulation of endosomal cholesterol [27]. We found that cellular entry of MERS-CoV was sensitive to inhibition by IFITMs, and observed that, at least in IFITM-transduced 293T cells, IFITM-dependent inhibition of human coronaviruses was more efficient than inhibition of emerging coronaviruses. However, the relative sensitivities to inhibition by IFITMs and endosomal cholesterol accumulation did not correlate. Finally, we obtained evidence that cellular entry of influenza A virus (FLUAV), which is IFITM-sensitive, cannot be blocked by certain compounds which induce endosomal cholesterol accumulation, suggesting that IFITMs can interfere with viral entry in a manner independent of endosomal cholesterol levels.

## 2. Results

**Figure 1:**
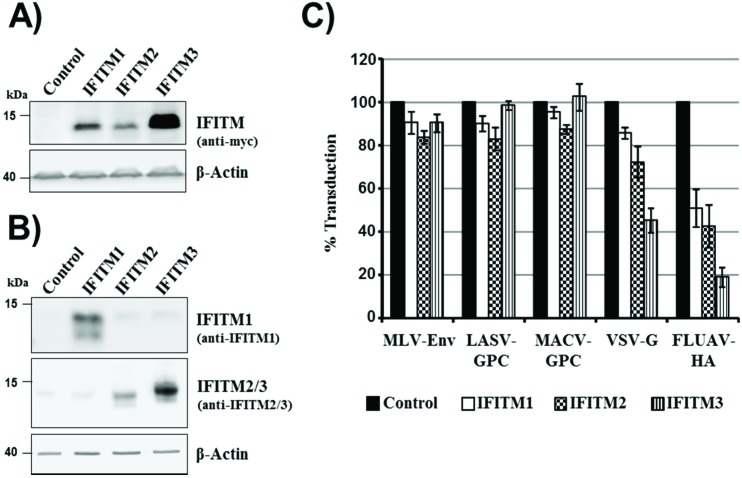
IFITM expression and antiviral activity. (**A**) 293T cells were transiently transduced with retroviral vectors encoding IFITM 1, 2 or 3 with a *C*-terminal c-myc tag or chloramphenicol acetyltransferase (cat) as control. Expression of IFITM proteins in cell lysates was determined by Western blot analysis, employing a myc-specific antibody. Expression of beta-actin was assessed as a loading control. Comparable results were obtained in a separate experiment; (**B**) The experiment was carried out as described for panel (A) but cells were transduced with vectors encoding IFITM proteins without antigenic tag, and IFITM expression was analyzed with an IFITM1-specific monoclonal antibody and an antiserum raised against IFITM2, which is cross-reactive with IFITM3. Similar results were obtained in an independent experiment (**C**) 293T cells, transiently transduced to express IFITM1, 2 or 3, or cat as described for panel (B), were transduced with infectivity-normalized MLV vectors encoding firefly luciferase and bearing the entry proteins of murine leukemia virus (MLV), Lassa virus (LASV), Machupo virus (MACV), vesicular stomatitis virus (VSV) or influenza A virus (FLUAV). At 72 h post inoculation, the transduction efficiency was determined by measuring luciferase activities in cell lysates. The average of four independent experiments, each carried out with triplicate samples, is shown. Error bars indicate standard error of the mean (SEM).

The embryonic kidney cell line 293T can be readily transfected and transduced and was, thus, chosen as the main tool for the present study. For directed expression of IFITM1, 2 and 3 in 293T cells, a retroviral vector system was employed. Western blot analysis of cells transduced with IFITM1, 2 and 3 variants, bearing a *C*-terminal c-myc tag, revealed robust expression of all IFITM proteins, although expression of IFITM3 was most efficient (Figure 1A). Similar results were obtained when cells transduced to express untagged IFITM proteins were analyzed, employing an IFITM1-specific antibody and an antiserum raised against IFITM2, which is cross-reactive with IFITM3 (Figure 1B). Moreover, this analysis revealed that endogenous expression of IFITM proteins in 293T cells was low or absent (Figure 1B). In order to test if the IFITM proteins unfold the expected antiviral activity, the IFITM-positive cells were transduced with retroviral vectors bearing the hemagglutinin (HA) of influenza A virus (FLUAV), the glycoprotein of the vesicular stomatitis virus (VSV-G), the envelope protein (Env) of murine leukemia virus (MLV) or the glycoproteins of Machupo virus (MACV-GPC) and Lassa virus (LASV-GPC). Cells expressing IFITM proteins without myc-tag were used for this experiment and all subsequent studies, since the tag might interfere with the antiviral activity of IFITMs. Expression of IFITM3 and to a lesser degree IFITM1 and 2 reduced entry driven by FLUAV-HA and VSV-G (Figure 1C), as expected [14]. In contrast, entry driven by MLV-Env, LASV-GPC or MACV-GPC was not inhibited (Figure 1C), again in keeping with published results [14], indicating that our experimental system was suitable for the analysis of the antiviral activity of IFITM proteins.

**Figure 2:**
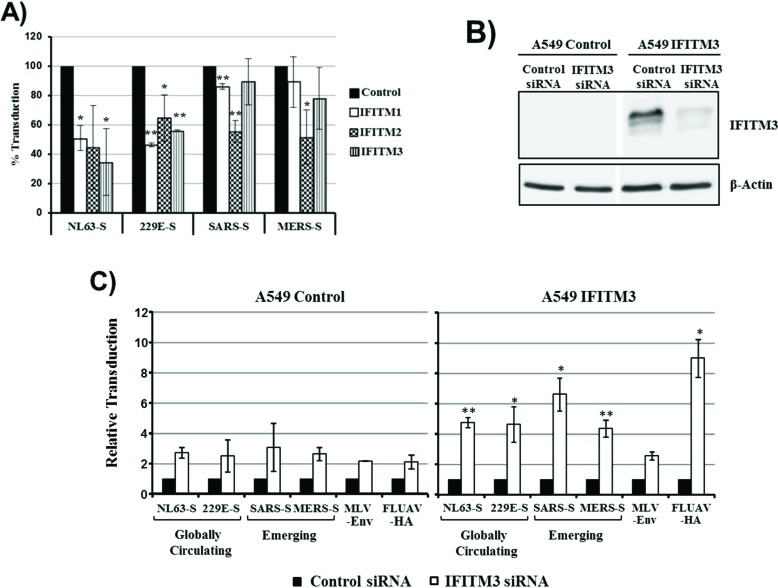
Inhibition of S protein-driven cell entry by IFITM proteins. (**A**) 293T cells, transfected to express the viral receptors and transduced to express IFITM1, 2, 3, or cat as control, were transduced with infectivity-normalized retroviral vectors bearing the S proteins of the globally circulating human coronaviruses NL63 and 229E as well as the S proteins of the emerging SARS- and MERS-CoV. Transduction efficiency was determined at 72 h post inoculation by measuring luciferase activity in cell lysates. Transduction of control cells was set as 100%. The average of three independent experiments carried out with triplicate samples is shown, error bars indicate SEM. Statistical significance was calculated using one tailed, paired *t*-test. * *p* ≤ 0.05; ** *p* ≤ 0.01; (**B**) A549 wild type cells (control) and A549 cells transduced to stably express IFITM3 were transfected with siRNA directed against IFITM3. Scrambled siRNA were used as a control. Knockdown of IFITM3 expression was analyzed by Western blot. Detection of β-actin served as a loading control; (**C**) A549 control cells or A549-IFITM3 cells were transfected with siRNA directed against IFITM3 or scrambled siRNA as control. Cells were then transduced with the retroviral vectors described in (A). Transduction efficiency was analyzed at 72 h post transduction. Transduction of cells transfected with the scrambled siRNA was set as 1. The average of three independent experiments performed with triplicate samples is shown; error bars indicate SEM. The Welch-Test for independent samples was used to determine whether the effects of the siRNAs on transduction of A549 control and A549-IFITM3 cells were significantly different. * *p* ≤ 0.05; ** *p* ≤ 0.01.

We next assessed whether the cellular entry driven by the S proteins of the human coronaviruses 229E and NL63 (229E-S, NL63-S) and the S proteins of the emerging coronaviruses SARS-CoV and MERS-CoV is differentially inhibited by IFITMs. Expression of all IFITM proteins reduced 229E-S- and NL63-S-driven entry about 40% to 70% (Figure 2). In contrast, IFITM1- and IFITM3-mediated inhibition of entry driven by SARS-S and MERS-S was inefficient (although in the case of SARS-S/IFITM1 statistically significant) and entry driven by these glycoproteins was only reduced to about 50% upon expression of IFITM2 (Figure 2). We next analyzed if the differential sensitivity to inhibition by IFITM proteins can also be detected in the context of a lung-derived cell line. For this, we engineered the human lung adenocarcinoma cell line A549 to stably express IFITM3 and analyzed the effects of an IFITM3-specific siRNA on S protein-mediated transduction. Transfection of parental A549 control cells with IFITM3 siRNA slightly increased transduction efficiency compared to cells transfected with scrambled, control siRNA. However, this effect was also observed with pseudotypes bearing the IFITM-insensitive MLV envelope protein and the IFITM-sensitive FLUAV-HA and was, thus, most likely unspecific. In contrast, transfection of A549-IFITM3 cells with IFITM3 siRNA efficiently increased HA- but not MLV-Env-driven transduction, indicating that an IFITM3-dependent block to viral entry was operative in these cells. Notably, entry into A549-IFITM3 cells driven by all S proteins studied was comparably augmented by IFITM3-specific siRNA, suggesting that the S proteins from globally circulating and emerging coronaviruses are comparably sensitive to inhibition by IFITM3 in this cellular system. In sum, 293T but not A549 cell entry driven by S proteins from human coronaviruses is more susceptible to inhibition by IFITM proteins than entry driven by the S proteins of emerging coronaviruses. Whether this differential IFITM sensitivity is due to differences in the expression levels of IFITM3, or other cellular components, remains to be determined.

**Figure 3:**
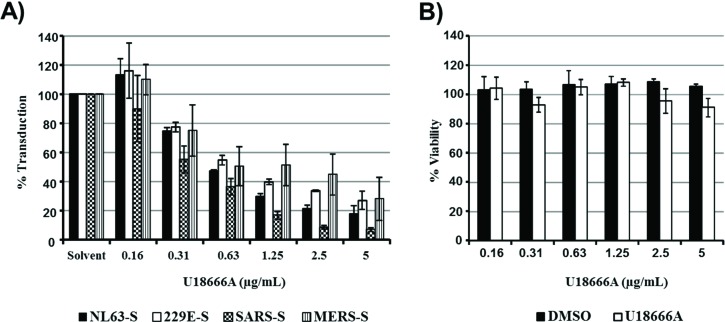
Sensitivity of S protein-driven entry to inhibition by IFITMs and U18666A do not correlate. (**A**) 293T cells expressing the viral receptors were treated with the indicated concentrations of U18666A, which increases endosomal cholesterol levels, and then transduced with retroviral particles bearing the indicated glycoproteins. Transduction efficiency was analyzed at 72 h post inoculation by determining luciferase activities in cell lysates. The average of two (NL63-S, 229E-S) to four (SARS-S, MERS-S) separate experiments carried out with triplicate samples are shown. Error bars indicate SEM. (**B**) Cytotoxicity of the indicated concentrations of U18666A or equal volumes of the solvent DMSO were measured under the conditions specified in (A) using an MTT reduction assay. The result of a single experiment carried out with triplicate samples is shown; error bars indicate standard deviation (SD). Similar results were obtained in two separate experiments.

It has been suggested that IFITMs inhibit viral entry into host cells by accumulating cholesterol within late endosomes [22]. We, therefore, investigated whether entry driven by the S proteins of globally circulating and emerging coronaviruses is differentially susceptible to inhibition by U18666A, a cationic amphiphilic drug, which induces the accumulation of cholesterol in late endosomes/lysosomes [27], and has been previously studied in the context of Ebola virus entry [28,29]. However, entry into 293T cells driven by the S proteins of the globally circulating hCoV-NL63 and 229E and the emerging MERS-CoV was comparably inhibited by U18666A, while inhibition of SARS-S-driven entry was most efficient (Figure 3A). Cytotoxic effects were not observed (Figure 3B). Thus, differential sensitivity to endosomal cholesterol might not account for the differential susceptibility of these S proteins to inhibition by IFITMs. 

**Figure 4:**
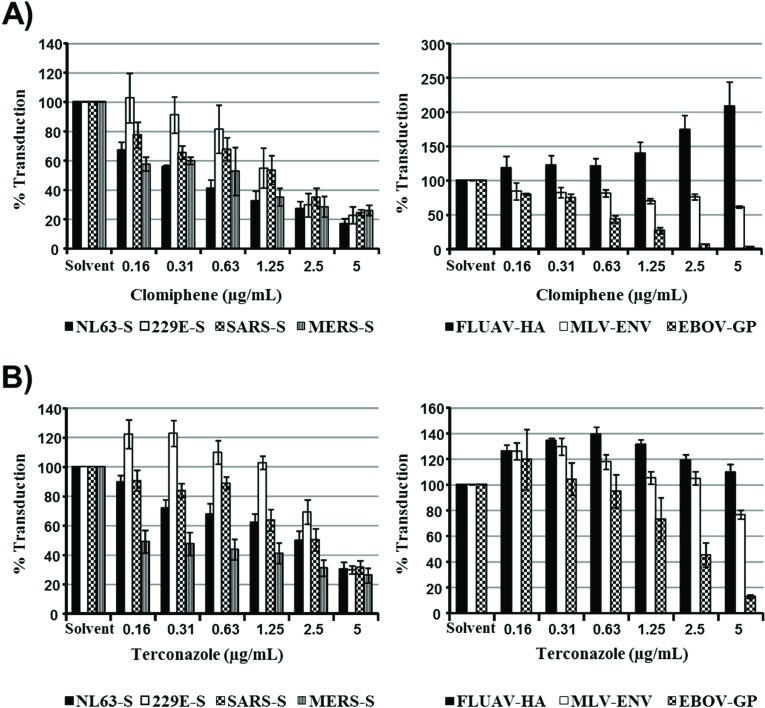
The relative sensitivities of coronavirus S protein- and FLUAV-HA-driven entry to inhibition by IFITMs and the cholesterol trafficking inhibitors clomiphene and terconazole do not correlate. 293T cells transfected to express the viral receptors were treated with the indicated concentrations of clomiphene (**A**) or terconazole (**B**), which increase endosomal cholesterol levels, and then transduced with retroviral particles bearing the indicated glycoproteins. Transduction efficiency was analyzed at 72 h post inoculation by determining luciferase activities in cell lysates. The average of three (clomiphene) or four (terconazole) independent experiments carried out with triplicate samples is shown. Error bars indicate SEM.

**Figure 5:**
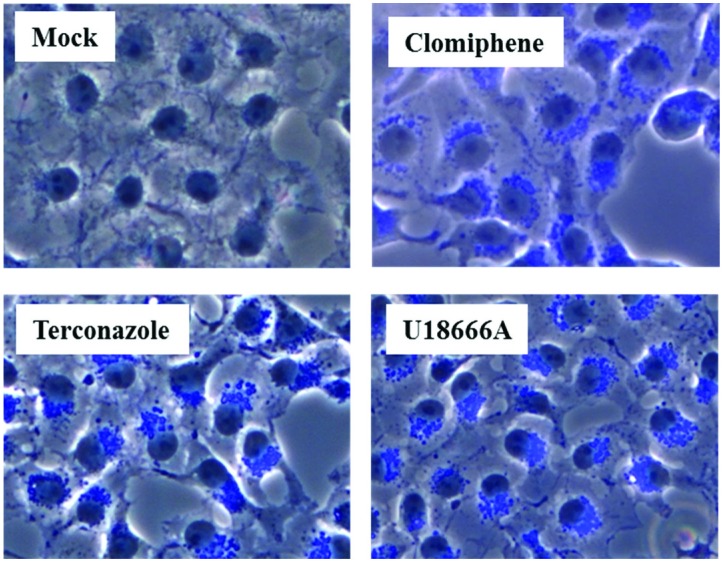
Cholesterol trafficking inhibitors induce accumulation of intracellular cholesterol. Cos7 cells were treated with either DMSO (mock) or the indicated inhibitors for 21 h at a concentration of 10 µM (terconazole, clomiphene) or 2.5 µM (U18666A). Cells were then fixed, stained with filipin III (blue) and staining analyzed by fluorescence microscopy using equal exposure times.

In order to further assess whether the sensitivity of viral entry to inhibition by IFITMs and increased cholesterol in late endosomes correlate, we investigated the impact of the cationic amphiphilic drugs clomiphene and terconazole on transduction efficiency, which, like U18666A, were found to induce cholesterol accumulation in late endosomes [29]. Both inhibitors reduced 293T cell entry driven by all coronavirus S proteins studied (Figure 4), and the relative inhibition efficiencies did not correlate with the sensitivities to inhibition by IFITMs, which were measured in the same cellular system (Figure 2). Moreover, although inhibitor sensitivity of MLV-Env and Zaire ebolavirus (EBOV)-GP-driven entry paralleled IFITM sensitivity, both clomiphene and terconazole either increased or had no effect on FLUAV-HA-dependent transduction (Figure 4), which is highly sensitive to inhibition by IFITM3 (Figure 1). Finally, filipin-staining of U18666A-, clomiphene- and terconazole-treated cells confirmed that these compounds indeed induced accumulation of cholesterol in intracellular compartments (Figure 5), as expected [29]. In sum, sensitivity of S protein- and, particularly, FLUAV-HA-driven entry to IFITM expression and endosomal cholesterol accumulation are not linked, indicating that modulation of cholesterol trafficking by IFITM proteins might not account for their ability to inhibit entry of certain viruses. 

## 3. Discussion

The IFITM proteins are IFN-induced effector molecules, which play an important role in the control of infection by FLUAV [23,24], RSV [25] and potentially other respiratory viruses. Here, we show that host cell entry driven by the S protein of the emerging, highly-pathogenic MERS-CoV is inhibited by IFITM proteins. In addition, we provide evidence that the S proteins of emerging coronaviruses might generally be less susceptible to inhibition by IFITM proteins than their counterparts in human coronaviruses, although these effects were dependent on the experimental system used. Moreover, we report that the relative susceptibilities of coronavirus S proteins to inhibition by IFITM proteins and compounds, which increase cholesterol levels in late endosomes [27], do not correlate. Moreover, entry driven by FLUAV-HA was insensitive to accumulation of endosomal cholesterol induced by some compounds despite high sensitivity to blockade by IFITM3. These observations identify MERS-CoV as a target for the antiviral activity of IFITM proteins and indicate that modulation of endosomal cholesterol levels might not be the only mechanism by which IFITM proteins can block viral entry.

For directed expression of IFITM proteins in target cells, we opted for retroviral transduction, which routinely allowed transgene expression in virtually all inoculated cells, as judged by employing a GFP-encoding vector (98.6% ± 0.7% GFP-positive cells, average of three independent experiments). Analysis of IFITM expression using an antibody directed against a *C*-terminal antigenic tag revealed that under the conditions chosen IFITM3 expression was most efficient while expression of IFITM1 and particularly IFITM2 was less robust. Similar results were obtained when expression of untagged proteins was determined, employing IFITM-specific antibodies. A more prominent expression of IFITM3 relative to IFITM1 and 2 in transduced 293T cells was also reported by a previous study [18], but the differences observed were less pronounced. Transduction of target cells with IFITM-encoding vectors consistently inhibited VSV-G- and FLUAV-HA-driven entry, with IFITM1 being least active and IFITM3 exerting the most prominent antiviral activity, as expected [14]. In contrast, entry driven by MLV-Env, MACV-GPC and LASV-GPC was not appreciably inhibited, again in keeping with published data [14]. Thus, the conditions established allowed assessment of the antiviral activity of IFITM proteins.

Expression of IFITM proteins in target cells inhibited entry driven by the S protein of MERS-CoV, indicating that IFITMs might contribute to the innate defenses against MERS-CoV invasion. This finding was not necessarily to be expected in light of a very recent report demonstrating that IFN treatment can increase host cell entry of the human coronavirus OC43 in an IFITM2/3-dependent fashion [30]. Comparison of S proteins from human and emerging coronaviruses for inhibition by IFITM proteins revealed consistent differences: Entry into 293T cells driven by the S proteins of hCoV-NL63 and hCoV-229E was uniformly and efficiently inhibited by expression of all IFITM proteins while entry driven by the S proteins of the emerging coronaviruses SARS-CoV and MERS-CoV was only appreciably reduced by expression of IFITM2, although it needs to be noted that IFITM1-dependent inhibition of SARS-S-driven transduction was statistically significant. In addition, the efficiency of entry inhibition by IFITM proteins depends on the expression level [17] and it cannot be excluded that blockade of SARS-S- and MERS-S-driven entry by IFITM proteins will be more prominent in cells expressing higher levels of these proteins. Nevertheless, the robust inhibition of 229E-S mediated entry by IFITM1, 2 and 3 is largely in keeping with a previous study [17]. Similarly, the finding that IFITM2 inhibits SARS-S-driven entry with markedly higher efficiency than IFITM1 and IFITM3 matches the results of a separate study [31]. In contrast, Huang and colleagues reported that IFITM1, 2 and 3 can all robustly reduce SARS-S-mediated transduction of Vero cells [18]. The reason for these discrepant findings is at present unclear. However, substantial inter-experiment variations in the relative inhibition of SARS-S-driven entry by IFITM1, 2 and 3 were observed in the previous study [18] and cell type-dependent differences in the antiviral activity of IFITM proteins might play a role. Such a scenario is supported by our observation that SARS-S and MERS-S-driven entry was not appreciably inhibited by IFITM3 expression in 293T cells while a reduction of IFITM3 expression in A549 cells notably augmented entry driven by all S proteins examined. Although these issues require further clarification with a particular focus on the relative expression levels of IFITM3 in transduced 293T and A549 cells, our results suggest that human coronaviruses (with the exception of hCoV-OC43) and emerging coronaviruses might be differentially susceptible to control by IFITM proteins, at least in certain target cells. Moreover, our observations are compatible with the concept that the antiviral activity of IFITM proteins might depend to some extent on the cellular context. Whether certain polymorphisms in the IFITM genes are associated with the severity of SARS or MERS, as reported for influenza [24], remains to be examined.

Expression of IFITM proteins can inhibit the host cell entry of many enveloped viruses [15]. A recent study provided insights into the mechanism underlying the broad antiviral activity of IFITM proteins. Amini-Bavil-Olyaee and colleagues reported that IFITM proteins bind, via their second transmembrane domain, to VAPA (vesicle-membrane-protein-associated protein A) and thereby interrupt the interaction of this protein with OSBP (oxysterol-binding protein), which is required for appropriate intracellular transport of cholesterol [22]. As a consequence of the disturbed interaction between VAPA and OSBP, cholesterol accumulates in late endosomal compartments and thereby blocks fusion of viral membranes with endosomal membranes [22]. The compounds U18666A, terconazole and clomiphene also induce accumulation of cholesterol in endosomal compartments [27,29] and were shown to inhibit EBOV entry into target cells [28,29]. Moreover, U18666A was found to interfere with FLUAV infection [32,33]. Our results show that the relative sensitivities of the S proteins of human and emerging coronaviruses to inhibition by IFITMs and to accumulation of endosomal cholesterol, induced by U18666A, terconazole and clomiphene, do not correlate. In addition, FLUAV-HA-driven entry was blocked by U18666A (98.9% ± 0.4% inhibition at 5 µg/mL U18666A, average of two independent experiments), as expected [32], but was not sensitive to inhibition by terconazole and clomiphene. These observations suggest that the inhibitory activity of U18666A was not due to the induction of cholesterol accumulation in late endosomes, in keeping with recently reported findings [21,34], but to another activity of U18666A – potentially the elevation of the endosomal pH [35]. Further studies are, therefore, required to elucidate how S protein- and HA-driven entry is blocked by IFITM proteins and direct effects of IFITMs on membrane fluidity and/or composition are one possibility [21,33,36].

## 4. Experimental Section

### 4.1. Cell Culture and Transfection

Human embryonic kidney 293T cells were maintained in Dulbecco’s minimal essential medium (DMEM), supplemented with 10% fetal bovine serum (FBS; PAA, Pasching, Austria; Biochrom, Berlin, Germany), 100 U/mL penicillin and 100 µg/mL streptomycin (Cytogen, Sinn, Germany). A549 cells stably expressing IFITM3 were created by transducing A549 wild type cells with the retroviral vector pQCXIP-IFITM3. Transduced cells were selected using the puromycin-resistance-cassette of the pQCXIP vector [14] and were maintained in DMEM containing FBS, penicillin/streptomycin and 2.5 µg puromycin/mL (Cayman chemical, Ann Arbor, MI, USA).

### 4.2. Plasmids

Plasmids encoding viral envelope proteins from vesicular stomatitis virus (VSV-G), murine leukemia virus (MLV-Env), Zaire ebolavirus (EBOV), Lassa virus (LASV-GPC), Machupo virus (MACV-GPC), influenza A virus (strain A/WSN/33 (FLUAV-HA)), severe acute respiratory syndrome coronavirus (SARS-S, Middle East respiratory syndrome coronavirus (MERS-S), human coronavirus NL63 (NL63-S) and human coronavirus 229E (229E-S) have been described previously [18,37,38,39,40,41]. Plasmids encoding the coronavirus receptors CD13 (used by hCoV-229E), CD26 (used by MERS-CoV) and ACE2 (used by hCoV-NL63 and SARS-CoV) have also been described previously [40,42]. Retroviral pQCXIP vectors for expression of IFITM1, 2 and 3, as well as MLV gag-pol and firefly-luciferase encoding MLV vector have also been described [14,32,43]. For construction of the control vector pQCXIP-Cat, the cat gene was PCR amplified from pDM128-CMV [44] and cloned into pENTR-2B-Dual (Invitrogen, Karlsruhe, Germany) via Acc65I and XhoI. Subsequently, the cat sequence was removed from pENTR-2B-Dual by NotI and BglII digest and inserted into pQCXIP previously digested with NotI and BamHI.

### 4.3. Western Blot

For immunoblotting, cells were lysed in Laemmli buffer and boiled for 10–30 min. Cell lysates were separated by SDS-PAGE and blotted onto nitrocellulose membranes. Membranes were blocked with 5% milk powder in PBS/0.1% Tween. Expression of myc-tagged IFITM proteins was analyzed with an antibody directed against the myc-tag [45]. Expression of untagged IFITM proteins was analyzed employing a monoclonal antibody specific for IFITM1 and a polyclonal IFITM2 antiserum, which is cross-reactive with IFITM3 (Proteintech, Chicago, IL, USA). As loading control, expression of β-actin was detected with murine anti β-actin antibody (Sigma-Aldrich, Deisenhofen, Germany). Bound antibodies were detected with horseradish peroxidase-(HRP-)-conjugated secondary antibody (Dianova, Hamburg, Germany) and luminescent substrate (Amersham, Amersham, UK).

### 4.4. Production of Retroviral Vectors

For the production of vectors encoding IFITMs, 293T cells were seeded in a T-75 cell culture flask at 60%–70% confluency and then cotransfected by calcium-phosphate-precipitation with plasmids encoding for MLV gag-pol (9 µg), VSV-G (9 µg) and pQCXIP-based vectors encoding IFITM proteins or cat as a control (18 µg). The culture medium was exchanged at 6 h post transfection and vector-containing supernatants were harvested at 48 h after transfection. The supernatants were cleared by filtration through a 0.45 µm filter and then stored at −80 °C. To produce firefly-luciferase encoding vectors bearing different viral glycoproteins, cells were seeded in T-25 cell culture flasks and then cotransfected with plasmids encoding for MLV gag-pol (3 µg), a firefly-luciferase harboring MLV vector (6 µg), and a plasmid encoding for the viral envelope protein (3 µg). Culture supernatants were harvested as described above, normalized for comparable transduction of untreated 293T cells and subsequently used for transduction experiments.

### 4.5. Transduction Experiments

To analyze inhibition of viral entry by IFITM proteins, 293T cells cotransfected to express CD26, ACE2 and CD13 were seeded at a density of 10^4^ cells per well in 96-well plates. Subsequently, preparations of IFITM encoding vectors were spinoculated [46] onto the cells by centrifugation at 4000× *g* for 30 min, followed by 48 h of incubation at 37 °C. Afterwards, the vector-containing supernatants were replaced by 50 µL of fresh culture medium. Thereafter, 50 µL of supernatants containing infectivity normalized, luciferase encoding vectors bearing different viral envelope proteins were added and the cells incubated for 8 h. Thereafter, the culture supernatants were replaced by 150 µL of fresh culture-medium and luciferase activity in cell lysates was measured at 72 h after transduction.

### 4.6. Inhibition of Transduction by Cationic Amphiphiles

To analyze the inhibition of virus glycoprotein-driven entry by the cationic amphiphiles, U18666A ((3β)-3-[2-(Diethylamino)ethoxy]androst-5-en-17-one hydrochloride) (Merck, Darmstadt, Germany, 662015), clomiphene (Sigma-Aldrich, C6272) or terconazole (Sigma-Aldrich, 32355), 293T cells transfected to express coronavirus receptors were seeded in 96-well plates at a density of 10^4^ cells per well. One hour before transduction the culture medium was removed and cells were treated with 50 µL of DMEM containing twice the final inhibitor concentration or DMSO as control. Thereafter, the cells were inoculated with 50 µL of vector preparations and incubated for 8 h at 37 °C. Finally, the transduction medium was replaced by 150 µL of fresh culture medium and luciferase activities in cell lysates were determined at 72 h post transduction.

### 4.7. Determination of Cytotoxicity

Cytotoxic effects of U18666A or DMSO to 293T cells were measured using a MTT (3-(4,5-dimethylthiazol-2-yl)-2,5-diphenyltetrazolium bromide) reduction assay [47]. 293T cells were preincubated with medium containing U18666A or DMSO at twice the final concentration. After 1 h the same volume of medium was added to reach the final inhibitor concentrations and the cells were subsequently incubated for 8 h in the presence of the inhibitor. Afterwards the medium was changed and 10 µL of MTT stock solution (5 mg/mL) were added. After incubation for 4 h at 37 °C, formazan crystals were dissolved by addition of 100 µL acidified isopropanol. Reduction of MTT was analyzed by measuring the absorption at 595 nm in an ELISA plate reader. Absorption of untreated cells was set to 100%.

### 4.8. Detection of Cholesterol Accumulation

The accumulation of intracellular cholesterol after treatment with cholesterol trafficking inhibitors was analyzed by fluorescence microscopy, using filipin as a dye. For this, 30,000 Cos7 cells per well were seeded in 24-well-plates containing glass cover slides. The next day, the cells were treated with the inhibitors for 21 h. Cells were then fixed with 4% PFA for 1 h at room temperature. Afterwards, cells were washed twice with PBS and then incubated in 50 µg/mL filipin III (Sigma-Aldrich) for 1 h at room temperature to stain for cholesterol. Subsequently, cells were washed three times with PBS, and then mounted with vectashield mounting medium (Vector laboratories, Burlingame, CA, USA). Microscopic analysis of the filipin staining was performed on an Axio Observer.A1 fluorescence microscope (Zeiss, Göttingen, Germany), and equal exposure times were used for imaging of cells treated with the three inhibitors tested.

### 4.9. siRNA Knockdown of IFITM3 Expression

To assess the effects of IFITM3 expression in A549 cells, wild type control cells and A549 cells stably transduced to express IFITM3 were transfected (Lipofectamine RNAiMAX, Invitrogen) with siRNAs directed against IFITM3 (Santa Cruz, Dallas, TX, USA; sc97053) or scrambled siRNA (Santa Cruz; sc37007) as a control. Cells were then analyzed by Western blot for IFITM3 expression, or transduced with pseudotyped MLV-particles, as described above. siRNA-mediated knock-down of IFITM3 expression was performed according to the manufacturer’s protocols. Shortly, 10,000 cells per well were seeded in a 96-well-plate. Cells were then transfected with 1.2 pmol siRNA and 0.2 µL Lipofectamine RNAiMAX per well. Transduction with pseudotypes or Western blot analysis of IFITM3 expression was performed at 48 h after siRNA transfection.

## 5. Conclusions

The present study shows that cellular entry driven by the S protein of the novel MERS-CoV is sensitive to inhibition by IFITM proteins. Moreover, evidence is presented that that human coronaviruses 229E and NL63 might be more susceptible to inhibition by IFITM proteins than the emerging SARS and MERS coronaviruses. Finally, our results demonstrate that mechanisms other than accumulation of endosomal cholesterol might be responsible for the inhibition of S protein- and FLUAV-HA-driven entry by IFITM proteins.

## References

[1] Holmes K.V., Knipe D. (2001). Coronaviruses. Fields Virology.

[2] Wevers B.A., van der Hoek L. (2009). Recently discovered human coronaviruses. Clin. Lab. Med..

[3] Chiu S.S., Chan K.H., Chu K.W., Kwan S.W., Guan Y., Poon L.L., Peiris J.S. (2005). Human coronavirus NL63 infection and other coronavirus infections in children hospitalized with acute respiratory disease in Hong Kong, China. Clin. Infect. Dis..

[4] Gorse G.J., O’Connor T.Z., Hall S.L., Vitale J.N., Nichol K.L. (2009). Human coronavirus and acute respiratory illness in older adults with chronic obstructive pulmonary disease. J. Infect. Dis..

[5] Jean A., Quach C., Yung A., Semret M. (2013). Severity and Outcome Associated with Human Coronavirus OC43 Infections among Children. Pediatr. Infect. Dis. J..

[6] Jevsnik M., Ursic T., Zigon N., Lusa L., Krivec U., Petrovec M. (2012). Coronavirus infections in hospitalized pediatric patients with acute respiratory tract disease. BMC Infect. Dis..

[7] Van der Hoek L., Sure K., Ihorst G., Stang A., Pyrc K., Jebbink M.F., Petersen G., Forster J., Berkhout B. (2005). Croup is associated with the novel coronavirus NL63. PLoS Med..

[8] Woo P.C., Lau S.K., Tsoi H.W., Huang Y., Poon R.W., Chu C.M., Lee R.A., Luk W.K., Wong G.K., Wong B.H. (2005). Clinical and molecular epidemiological features of coronavirus HKU1-associated community-acquired pneumonia. J. Infect. Dis..

[9] Graham R.L., Donaldson E.F., Baric R.S. (2013). A decade after SARS: Strategies for controlling emerging coronaviruses. Nat. Rev. Microbiol..

[10] Peiris J.S., Guan Y., Yuen K.Y. (2004). Severe acute respiratory syndrome. Nat. Med..

[11] Stadler K., Rappuoli R. (2005). SARS: Understanding the virus and development of rational therapy. Curr. Mol. Med..

[12] WHO (2014). Summary of Probable SARS Cases with Onset of Illness from 1 November 2002 to 31 July 2003.

[13] WHO (2014). Middle East Respiratory Syndrome Coronavirus (MERS-CoV)-Update.

[14] Brass A.L., Huang I.C., Benita Y., John S.P., Krishnan M.N., Feeley E.M., Ryan B.J., Weyer J.L., van der Weyden L., Fikrig E. (2009). The IFITM proteins mediate cellular resistance to influenza A H1N1 virus, West Nile virus, and dengue virus. Cell.

[15] Perreira J.M., Chin C.R., Feeley E.M., Brass A.L. (2013). IFITMs restrict the replication of multiple pathogenic viruses. J. Mol. Biol..

[16] Schoggins J.W., Wilson S.J., Panis M., Murphy M.Y., Jones C.T., Bieniasz P., Rice C.M. (2011). A diverse range of gene products are effectors of the type I interferon antiviral response. Nature.

[17] Bertram S., Dijkman R., Habjan M., Heurich A., Gierer S., Glowacka I., Welsch K., Winkler M., Schneider H., Hofmann-Winkler H. (2013). TMPRSS2 activates the human coronavirus 229E for cathepsin-independent host cell entry and is expressed in viral target cells in the respiratory epithelium. J. Virol..

[18] Huang I.C., Bailey C.C., Weyer J.L., Radoshitzky S.R., Becker M.M., Chiang J.J., Brass A.L., Ahmed A.A., Chi X., Dong L. (2011). Distinct patterns of IFITM-mediated restriction of filoviruses, SARS coronavirus, and influenza A virus. PLoS Pathog..

[19] Feeley E.M., Sims J.S., John S.P., Chin C.R., Pertel T., Chen L.M., Gaiha G.D., Ryan B.J., Donis R.O., Elledge S.J. (2011). IFITM3 inhibits influenza A virus infection by preventing cytosolic entry. PLoS Pathog..

[20] Li K., Markosyan R.M., Zheng Y.M., Golfetto O., Bungart B., Li M., Ding S., He Y., Liang C., Lee J.C. (2013). IFITM proteins restrict viral membrane hemifusion. PLoS Pathog..

[21] Desai T.M., Marin M., Chin C.R., Savidis G., Brass A.L., Melikyan G.B. (2014). IFITM3 restricts influenza A virus entry by blocking the formation of fusion pores following virus-endosome hemifusion. PLoS Pathog..

[22] Amini-Bavil-Olyaee S., Choi Y.J., Lee J.H., Shi M., Huang I.C., Farzan M., Jung J.U. (2013). The antiviral effector IFITM3 disrupts intracellular cholesterol homeostasis to block viral entry. Cell Host. Microbe.

[23] Bailey C.C., Huang I.C., Kam C., Farzan M. (2012). Ifitm3 limits the severity of acute influenza in mice. PLoS Pathog..

[24] Everitt A.R., Clare S., Pertel T., John S.P., Wash R.S., Smith S.E., Chin C.R., Feeley E.M., Sims J.S., Adams D.J. (2012). IFITM3 restricts the morbidity and mortality associated with influenza. Nature.

[25] Everitt A.R., Clare S., McDonald J.U., Kane L., Harcourt K., Ahras M., Lall A., Hale C., Rodgers A., Young D.B. (2013). Defining the range of pathogens susceptible to Ifitm3 restriction using a knockout mouse model. PLoS One.

[26] Mills T.C., Rautanen A., Elliott K.S., Parks T., Naranbhai V., Ieven M.M., Butler C.C., Little P., Verheij T., Garrard C.S. (2013). IFITM3 and susceptibility to respiratory viral infections in the community. J. Infect. Dis..

[27] Cenedella R.J. (2009). Cholesterol synthesis inhibitor U18666A and the role of sterol metabolism and trafficking in numerous pathophysiological processes. Lipids.

[28] Carette J.E., Raaben M., Wong A.C., Herbert A.S., Obernosterer G., Mulherkar N., Kuehne A.I., Kranzusch P.J., Griffin A.M., Ruthel G. (2011). Ebola virus entry requires the cholesterol transporter Niemann-Pick C1. Nature.

[29] Shoemaker C.J., Schornberg K.L., Delos S.E., Scully C., Pajouhesh H., Olinger G.G., Johansen L.M., White J.M. (2013). Multiple cationic amphiphiles induce a Niemann-Pick C phenotype and inhibit Ebola virus entry and infection. PLoS One.

[30] Zhao X., Guo F., Liu F., Cuconati A., Chang J., Block T.M., Guo J.T. (2014). Interferon induction of IFITM proteins promotes infection by human coronavirus OC43. Proc. Natl. Acad. Sci. USA.

[31] Warren C.J., Griffin L.M., Little A.S., Huang I.C., Farzan M., Pyeon D. (2014). The antiviral restriction factors IFITM1, 2 and 3 do not inhibit infection of human papillomavirus, cytomegalovirus and adenovirus. PLoS One.

[32] Eckert N., Wrensch F., Gartner S., Palanisamy N., Goedecke U., Jager N., Pöhlmann S., Winkler M. (2014). Influenza a virus encoding secreted gaussia luciferase as useful tool to analyze viral replication and its inhibition by antiviral compounds and cellular proteins. PLoS One.

[33] Musiol A., Gran S., Ehrhardt C., Ludwig S., Grewal T., Gerke V., Rescher U. (2013). Annexin A6-balanced late endosomal cholesterol controls influenza a replication and propagation. MBio.

[34] Lin T.Y., Chin C.R., Everitt A.R., Clare S., Perreira J.M., Savidis G., Aker A.M., John S.P., Sarlah D., Carreira E.M. (2013). Amphotericin B increases influenza A virus infection by preventing IFITM3-mediated restriction. Cell Rep..

[35] Lafourcade C., Sobo K., Kieffer-Jaquinod S., Garin J., van der Goot F.G. (2008). Regulation of the V-ATPase along the endocytic pathway occurs through reversible subunit association and membrane localization. PLoS One.

[36] John S.P., Chin C.R., Perreira J.M., Feeley E.M., Aker A.M., Savidis G., Smith S.E., Elia A.E., Everitt A.R., Vora M. (2013). The CD225 domain of IFITM3 is required for both IFITM protein association and inhibition of influenza A virus and dengue virus replication. J. Virol..

[37] Chaipan C., Kobasa D., Bertram S., Glowacka I., Steffen I., Tsegaye T.S., Takeda M., Bugge T.H., Kim S., Park Y. (2009). Proteolytic activation of the 1918 influenza virus hemagglutinin. J. Virol..

[38] Gierer S., Bertram S., Kaup F., Wrensch F., Heurich A., Kramer-Kuhl A., Welsch K., Winkler M., Meyer B., Drosten C. (2013). The spike protein of the emerging betacoronavirus EMC uses a novel coronavirus receptor for entry, can be activated by TMPRSS2, and is targeted by neutralizing antibodies. J. Virol..

[39] Hofmann H., Hattermann K., Marzi A., Gramberg T., Geier M., Krumbiegel M., Kuate S., Uberla K., Niedrig M. (2004). S protein of severe acute respiratory syndrome-associated coronavirus mediates entry into hepatoma cell lines and is targeted by neutralizing antibodies in infected patients. J. Virol..

[40] Hofmann H., Pyrc K., van der Hoek L., Geier M., Berkhout B., Pöhlmann S. (2005). Human coronavirus NL63 employs the severe acute respiratory syndrome coronavirus receptor for cellular entry. Proc. Natl. Acad. Sci. USA.

[41] Simmons G., Reeves J.D., Grogan C.C., Vandenberghe L.H., Baribaud F., Whitbeck J.C., Burke E., Buchmeier M.J., Soilleux E.J. (2003). DC-SIGN and DC-SIGNR bind ebola glycoproteins and enhance infection of macrophages and endothelial cells. Virology.

[42] Raj V.S., Mou H., Smits S.L., Dekkers D.H., Muller M.A., Dijkman R., Muth D., Demmers J.A., Zaki A., Fouchier R.A. (2013). Dipeptidyl peptidase 4 is a functional receptor for the emerging human coronavirus-EMC. Nature.

[43] Bartosch B., Dubuisson J., Cosset F.L. (2003). Infectious hepatitis C virus pseudo-particles containing functional E1-E2 envelope protein complexes. J. Exp. Med..

[44] Malim M.H., McCarn D.F., Tiley L.S., Cullen B.R. (1991). Mutational definition of the human immunodeficiency virus type 1 Rev activation domain. J. Virol..

[45] Evan G.I., Lewis G.K., Ramsay G., Bishop J.M. (1985). Isolation of monoclonal antibodies specific for human c-myc proto-oncogene product. Mol. Cell Biol..

[46] O’Doherty U., Swiggard W.J., Malim M.H. (2000). Human immunodeficiency virus type 1 spinoculation enhances infection through virus binding. J. Virol..

[47] Kupcsik L. (2011). Estimation of cell number based on metabolic activity: The MTT reduction assay. Methods Mol. Biol..

